# Upper Gastrointestinal Bleeding Secondary to Gastric Invasion by Diffuse Large B-cell Lymphoma

**DOI:** 10.7759/cureus.94594

**Published:** 2025-10-14

**Authors:** Helen Paglia, Marco Noriega, Kinnari R Kher

**Affiliations:** 1 Internal Medicine, Memorial Healthcare, Savannah, USA; 2 Internal Medicine, Mount Auburn Hospital, Harvard Medical School, Cambridge, USA; 3 Gastroenterology, Mount Auburn Hospital, Harvard Medical School, Cambridge, USA

**Keywords:** diffuse large b-cell lymphoma, gastric ulcer, hematemesis, hemostasis, lymphoma

## Abstract

Diffuse large B-cell lymphoma (DLBCL) is the most common non-Hodgkin lymphoma subtype. We present a case of an 80-year-old male with upper gastrointestinal bleeding (UGIB), ultimately diagnosed with DLBCL involving the liver, gallbladder, inferior vena cava, and stomach, leading to a large gastric ulcer. Despite initial suspicion of alcohol-related UGIB, imaging revealed multiple masses with extranodal lymphoma involvement. Endoscopic hemostasis was attempted but was complicated by tumor-induced neovascularization. This case highlights a rare cause of UGIB and underscores the challenges in diagnosis and management of malignancy-induced bleeding.

## Introduction

Upper gastrointestinal bleeding (UGIB) is a common and potentially life-threatening condition that can arise from various etiologies, including peptic ulcer disease (PUD), esophageal varices, and malignancy [1,2]. We present the case of a patient who presented to the emergency department with UGIB and was ultimately diagnosed with diffuse large B-cell lymphoma (DLBCL) involving the liver, gallbladder, inferior vena cava (IVC), and stomach, resulting in a large gastric ulcer [3]. This case emphasizes the importance of considering extranodal lymphoma as a potential cause of UGIB and highlights the diagnostic and therapeutic challenges associated with such rare presentations, including endoscopic hemostatic management and indications for imaging to evaluate further [4,5].

## Case presentation

An 80-year-old man with a past medical history significant for sleep apnea, essential hypertension, thyroid nodules, gait disorder, paresthesias, concussion, and arthritis presented to the ED in 2024 with emesis that began two to three days ago. It started as a light-colored substance and gradually became a coffee-ground-like emesis. Prior to developing emesis, the patient had experienced dark stools, fatigue, and lower extremity edema for two weeks. Two months prior to this, he noticed progressive onset of malaise, fatigue, an unspecified amount of weight loss, and night sweats. On presentation to the ED, he was tachycardic and had heme-positive coffee-ground-like emesis. In the ED, he was hemodynamically stable, and laboratory testing showed a hemoglobin of 4.9 g/dL and hematocrit of 15.9. His mean corpuscular volume (MCV) was normal at 88.3 fL, mean corpuscular hemoglobin concentration (MCHC) was low at 30.8 g/dL, red cell distribution width (RDW) was elevated at 15.8%, and the red blood cell (RBC) count was low at 1.8 x10^6 per microliter (Table 1).

**Table 1:** Laboratory findings of the patient presenting with UGIB due to DLBCL. UGIB: upper gastrointestinal bleeding; DLBCL: diffuse large B-cell lymphoma; MCV: mean corpuscular volume; MCHC: mean corpuscular hemoglobin concentration; RDW: red cell distribution width; RBC: red blood cell count; ALT: alanine aminotransferase; AST: aspartate aminotransferase; ALP: alkaline phosphatase

Lab Test	Patient Value	Normal Range
Hemoglobin	4.9 g/dL	12-16 g/dL
Hematocrit	15.90%	36-46%
MCV	88.3 fL	80-100 fL
MCHC	30.8 g/dL	32-36 g/dL
RDW	15.80%	11-15%
RBC	1.8 x10^6/µL	4.2-5.4 x10^6/µL
ALT	65 U/L	7-56 U/L
AST	72 U/L	10-40 U/L
ALP	110 U/L	44-147 U/L
Total bilirubin	1.2 mg/dL	0.1-1.2 mg/dL

The emergency medical technician (EMT) report noted multiple bottles of alcohol in his home, and therefore, alcohol-related UGIB was initially suspected. An urgent ultrasound (US) hepatic segment was done in the ED to rule out possible cirrhosis. The US scan showed an 11.7 x 11.5 x 9 cm heterogeneous hypoechoic solid mass within the right hepatic lobe with nodular contour and internal color doppler flow adjacent to the gallbladder fossa. Intrahepatic and extrahepatic ducts were normal. The gallbladder also showed a 3.7 x 3.4 x 2.4 heterogeneous hypoechoic solid mass without internal Doppler flow. There was a loss of the fat planes between the right hepatic lobe and the adjacent gallbladder.

**Figure 1:**
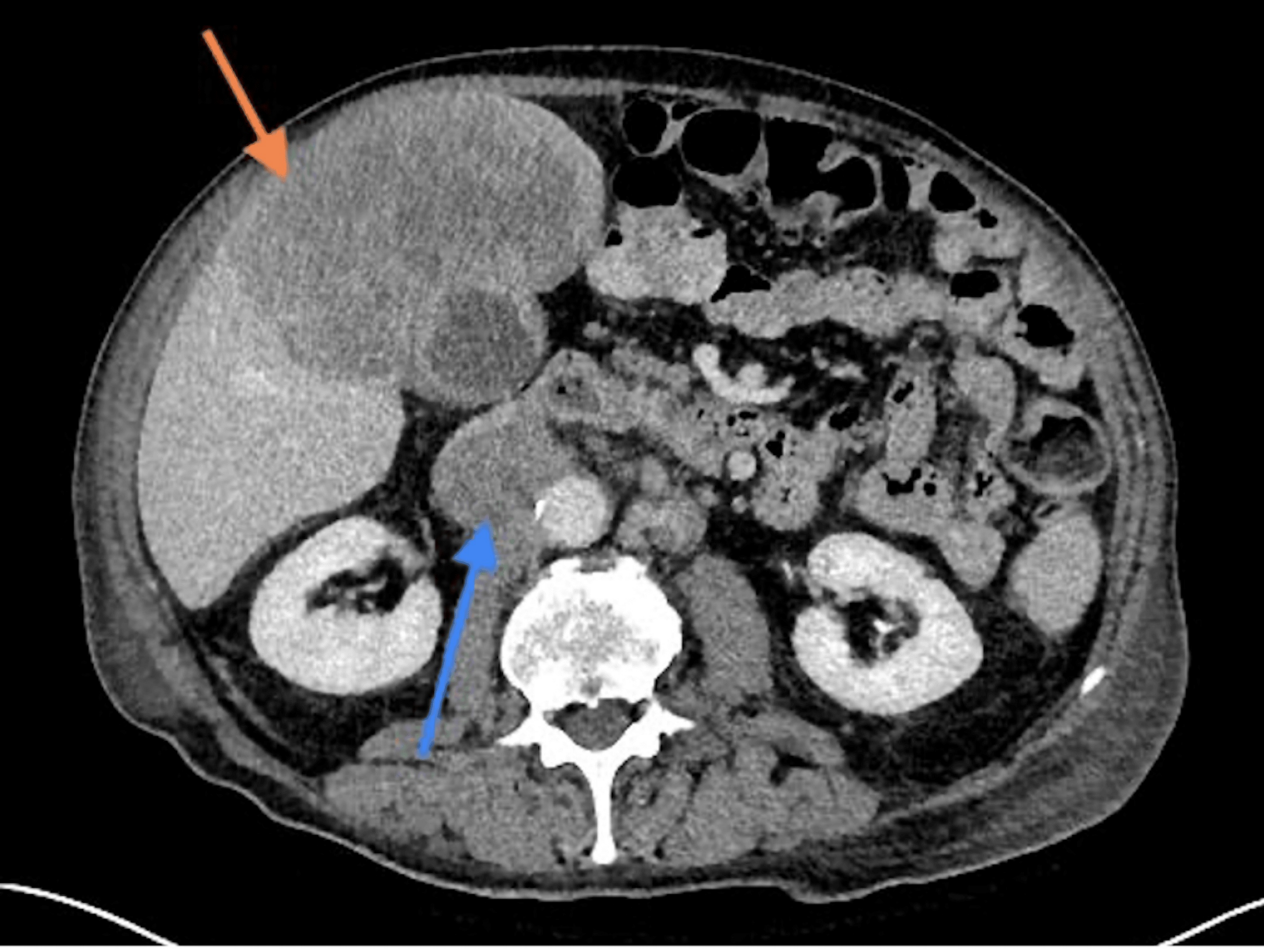
CT abdomen, transverse section. Orange arrow: mass surrounding the gallbladder; Blue arrow: inferior vena cava (IVC) tumor thrombus.

**Figure 2:**
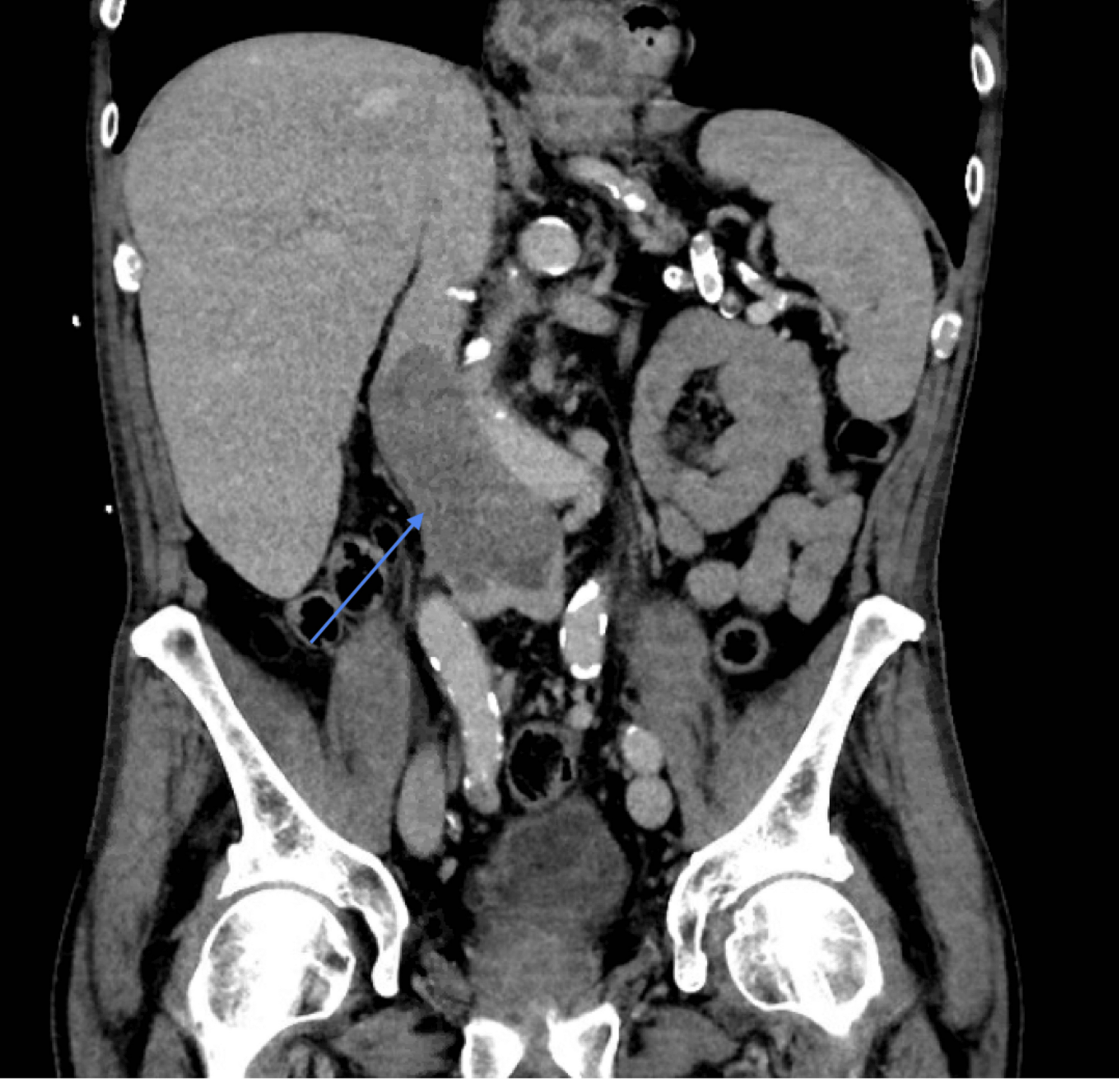
CT abdomen, coronal section. Blue arrow: inferior vena cava (IVC) tumor thrombus.

The patient underwent a CT scan of the abdomen and pelvis to evaluate the mass seen on US in the setting of a UGIB. These showed a large heterogeneous enhancing mass in hepatic segment 4, corresponding to the mass seen on US, effacing the fascial planes of the anterior and right lateral wall of the gallbladder. A large thrombus measuring 5.4 × 4.7 cm was also noted in the proximal IVC and was strongly suspected to be a tumor thrombus (Figures 1-2).

**Figure 3:**
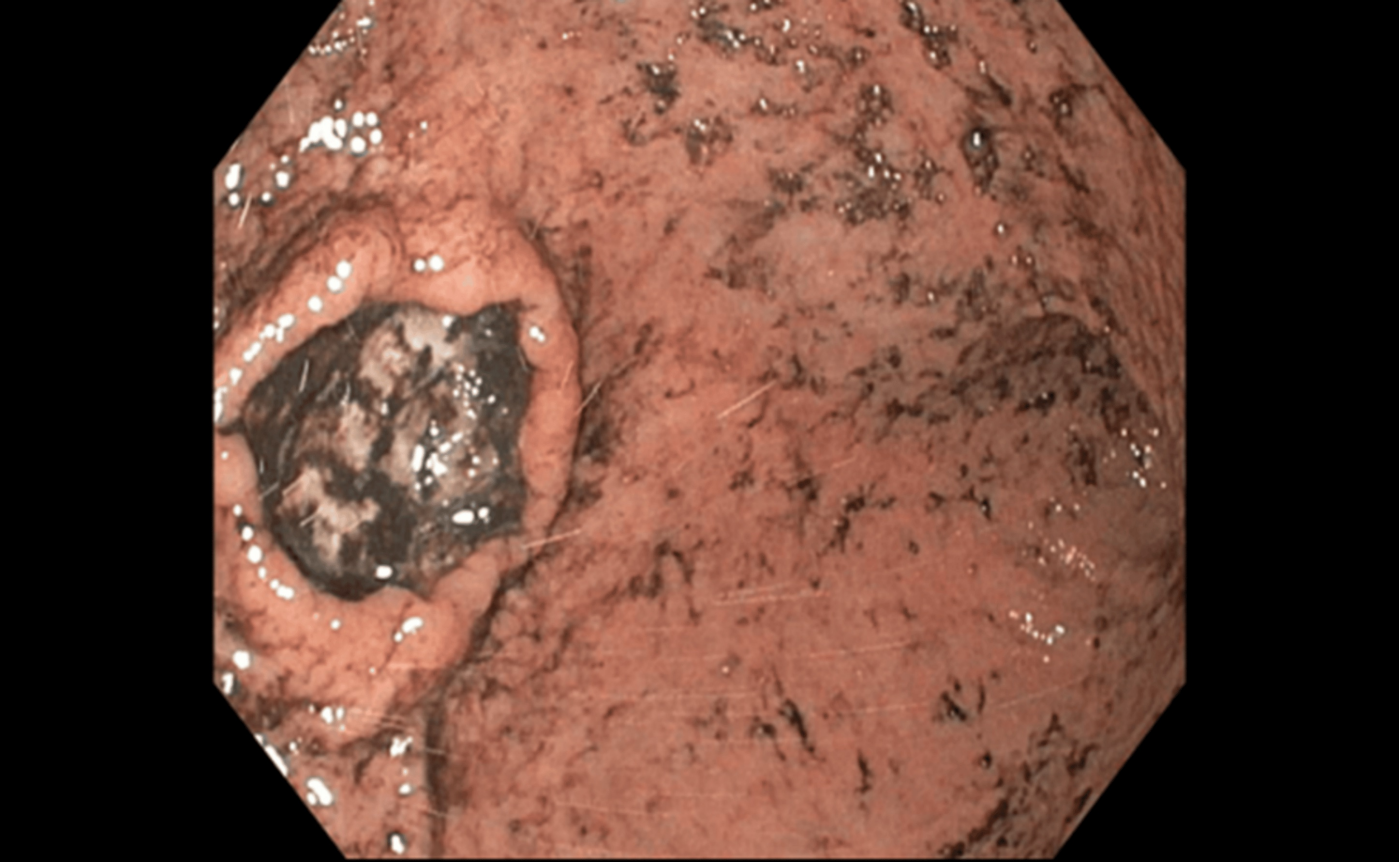
Ulcer on the greater curvature with coffee-ground material at the ulcer base.

An upper endoscopy (esophagogastroduodenoscopy, or EGD) showed a large clean-based ulcer on the greater curvature of the stomach, 3 cm in diameter, with rolled-up margins. The base of the ulcer was covered with coffee grounds, but there were no stigmata of bleeding noted (Figure 3). Application of a MANTIS Clip (Boston Scientific, Marlborough, MA, USA) was attempted, but the mucosa was tethered to the deeper layers, and the clip could not be successfully deployed.

**Figure 4:**
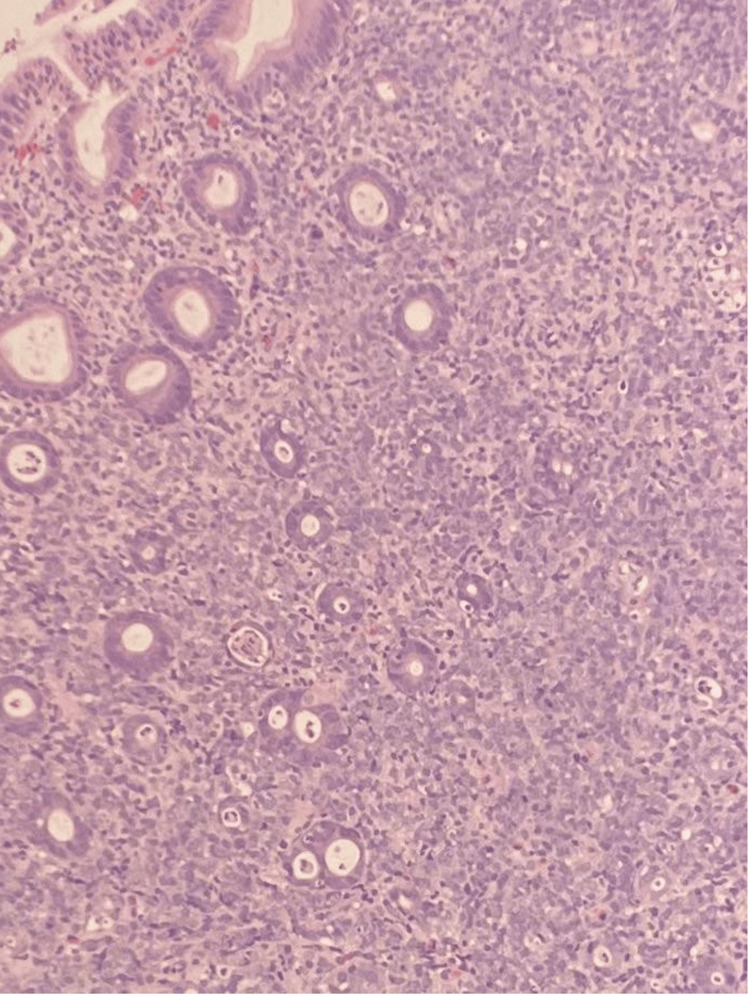
Low-power view of gastric mucosa showing lamina propria with gastric glands being replaced by sheets of lymphocytes and some lymphoepithelial lesions.

The patient’s bleeding stabilized, and the liver mass was biopsied four days later by interventional radiology, making the diagnosis of DLBCL evolving on the background of follicular lymphoma (Figure 4).

**Figure 5:**
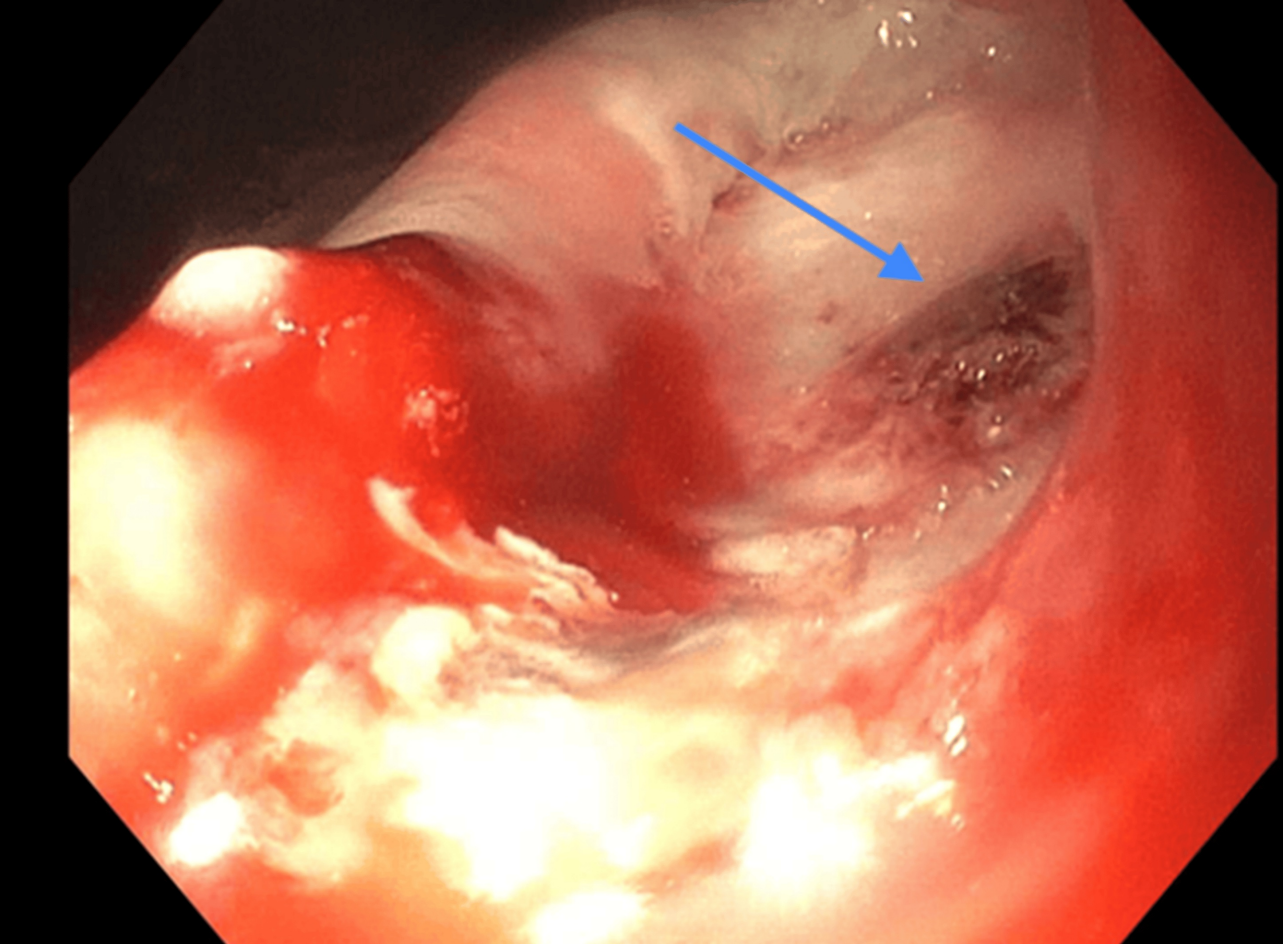
Large cratered gastric ulcer with a visible vessel in one area (blue arrow).

This biopsy was followed by another episode of melena and hypotension, requiring repeat EGD, which showed a large cratered gastric ulcer with a visible vessel in one area and an adherent clot in another. These areas were treated with a combination of bipolar cautery, 1:10,000 epinephrine injection, and Hemospray (Cook Medical, Bloomington, IN, USA) (Figure 5).

## Discussion

UGIB is a common and potentially life-threatening condition [1]. PUD is the most common cause of UGIB requiring hospitalization, accounting for approximately half of all cases [2,3]. However, rates of hospitalization due to PUD have declined over the last several decades since the inception of proton pump inhibitors (PPIs), whereas rarer causes, including Dieulafoy lesions, arteriovenous malformations, and malignancy, have been on the rise [4,5]. Malignancy is a rare cause of acute UGIB, accounting for only 1-5% of cases. Most malignant UGIB are due to primary gastric neoplasms, with metastatic disease being even less common [6-9].

Extranodal non-Hodgkin lymphoma (NHL) causing UGIB is a rare entity. DLBCL, the most common subtype of NHL, can involve extranodal sites, leading to clinical presentations that often mimic more common gastrointestinal disorders [9,10]. When lymphoma involves the gastrointestinal tract, it can cause direct invasion of the mucosa, resulting in ulcers and bleeding, as seen in this case. Therefore, large gastric ulcers (3 cm or larger) in the stomach should be considered for a CT scan to rule out lymphoma as a potential underlying cause. DLBCL's involvement of structures like the liver, gallbladder, and IVC may further complicate the clinical picture, requiring a high index of suspicion for accurate diagnosis and effective management [11,12].

Endoscopic hemostasis is an important technique for managing UGIB, particularly in patients with malignancies. When managing bleeding associated with malignant disease, it is important to consider tumor invasiveness, the possibility of necrosis, and the presence of ulceration. In this case, clip placement was attempted during endoscopy, but was not successful because the mucosa was tethered to the submucosa. The management of gastrointestinal bleeding in malignant disease presents unique challenges due to the multifocal nature of the bleeding caused by tumor-induced neovascularization, which leads to an increased number of abnormally structured blood vessels that can become exposed, making these tumors a significant source of gastrointestinal bleeding. Hemostatic spray is particularly advantageous in cases of diffuse or multifocal bleeding [13] when cautery and clips cannot be extensively deployed.

## Conclusions

This case highlights the importance of considering malignancy, particularly lymphoma, in the differential diagnosis of UGIB, especially when ulcers are large, atypical in appearance, or associated with systemic findings. Early cross‑sectional imaging and tissue diagnosis are essential for accurate identification of underlying pathology. Additionally, malignant ulcers pose unique challenges to endoscopic hemostasis, underscoring the need for a multimodal approach to both diagnosis and management.
